# Enhancing Membrane Materials for Efficient Li Recycling and Recovery

**DOI:** 10.1002/adma.202402335

**Published:** 2024-12-15

**Authors:** Xingpeng Tian, Chunchun Ye, Liyuan Zhang, Manoj K. Sugumar, Yan Zhao, Neil B. McKeown, Serena Margadonna, Rui Tan

**Affiliations:** ^1^ Warwick Electrochemical Engineering WMG University of Warwick Coventry CV4 7AL UK; ^2^ EaStChem School of Chemistry University of Edinburgh Edinburgh EH9 3FJ UK; ^3^ School of Metallurgy and Environment Central South University Changsha 410083 P. R. China; ^4^ School of Energy and Power Engineering Jiangsu University Zhenjiang 212013 China; ^5^ Department of Chemical Engineering Swansea University Swansea SA1 8EN UK

**Keywords:** lithium extraction and recovery, lithium transport mechanisms, membranes, separation

## Abstract

Rapid uptake of lithium‐centric technology, e.g., electric vehicles and large‐scale energy storage, is increasing the demand for efficient technologies for lithium extraction from aqueous sources. Among various lithium‐extraction technologies, membrane processes hold great promise due to energy efficiency and flexible operation in a continuous process with potential commercial viability. However, membrane separators face challenges such as the extraction efficiency due to the limited selectivity toward lithium relative to other species. Low selectivity can be ascribed to the uncontrollable selective channels and inefficient exclusion functions. However, recent selectivity enhancements for other membrane applications, such as in gas separation and energy storage, suggest that this may also be possible for lithium extraction. This review article focuses on the innovations in the membrane chemistries based on rational design following separation principles and unveiling the theories behind enhanced selectivity. Furthermore, recent progress in membrane‐based lithium extraction technologies is summarized with the emphasis on inorganic, organic, and composite materials. The challenges and opportunities for developing the next generation of selective membranes for lithium recovery are also pointed out.

## Graphical abstract

This review article systematically explores the recent advances in the membrane processes for lithium cycling and recovery, offering the first correlation between these technologies and technology readiness levels, unveiling the fundamentals for the lithium separation, and providing insights into the future design of membrane materials from both academic and industrial perspectives.

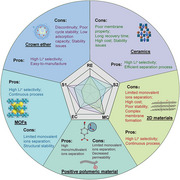

## 1 Overview

**Figure 1:**
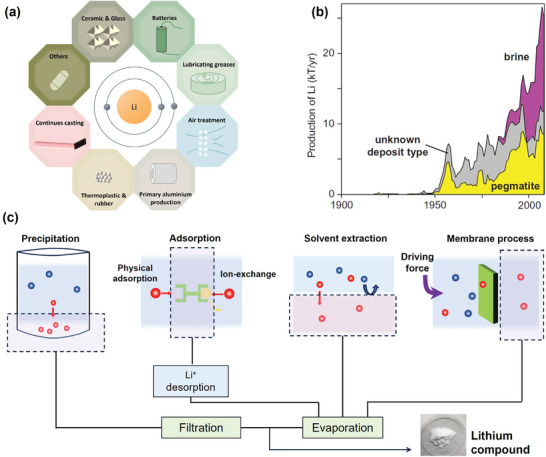
Overview of Li resources, applications, and recycling approaches. a) Lithium applications in various industries. b) Lithium extraction from different sources for the past century. Reproduced with permission.^[^ ^24^ ^]^ Copyright 2016, Society of Economic Geologists. c) Water‐based lithium recovery techniques, including precipitation, adsorption, solvent extraction, and membrane process. Red spheres: target recovery ions; blue spheres: other species.

Lithium has been widely investigated and applied in industries such as medicine, metallurgy, aerospace, and energy storage^[^ ^1^, ^2^, ^3^, ^4^, ^5^, ^6^ ^]^ (**Figure** **1**a). Rapid innovations in electrochemical storage systems (ESSs) have boosted the development of electrical vehicles and portable devices, expanding the demand for lithium resources.^[^ ^7^ ^]^ To date, lithium‐focused energy storage system has accounted for over half of the total lithium usage and continues to maintain an annual growth rate of ≈32%. By 2030, annual global lithium demand is projected to surpass two million tonnes of lithium carbonate equivalent.^[^ ^7^ ^]^ Hence, there is an urgent need to improve existing lithium extraction technologies and to develop new methods that provide greater efficiency.

In nature, lithium resources mainly exist in rocks, such as clays^[^ ^8^ ^]^ and pegmatites,^[^ ^9^ ^]^ and water resources mainly salt lake brines,^[^ ^10^ ^]^ oilfield brines,^[^ ^11^ ^]^ geothermal brines,^[^ ^12^ ^]^ and seawater.^[^ ^13^ ^]^ To date, 80 million tonnes of natural lithium resources have already been discovered, with over 90% of commercial lithium being currently supplied by Australia, Chile, Argentina, and China.^[^ ^14^ ^]^ While the development of lithium extraction from water resources occurred relatively later, lithium derived from brine currently accounts for more than half of the total annual production (Figure 1b). Continental brine, geothermal water, and even as yet untapped seawater all contain abundant lithium resources, making it a promising solution to meet future demand.^[^ ^15^ ^]^ Moreover, secondary lithium sources, represented by spent batteries, are growing dramatically in the foreseeable, which, in addition to substantial waste of resource, leads to a significant challenge for the environment. Although existing recovery processes, such as pyrometallurgical and hydrometallurgical methods, can achieve high lithium recoveries (>90%) for most lithium‐based electrode materials, significant limitations still exist in terms of cost‐effectiveness and economic viability, which include traditional methods of separating lithium from other metallic elements in the aqueous phase. Therefore, the development of advanced technologies to directly or assist in the battery recycling process has garnered widespread attention in recent years.^[^ ^16^, ^17^, ^18^ ^]^

**Figure 2:**
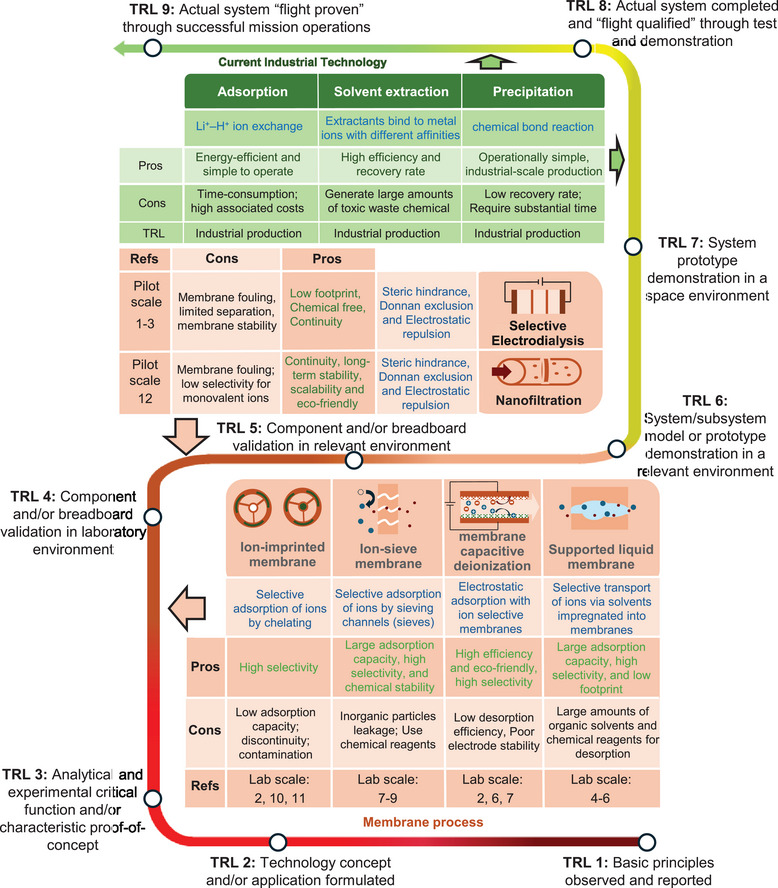
State‐of‐the‐art membrane‐based technologies for lithium recovery from aqueous environment. The assessment of TRL follows the standards set by National Aeronautics and Space Adminstration's management instruction.^[^ ^40^, ^41^ ^]^

The primary task of lithium extraction in the aqueous phase is the separation of different ions. A variety of aqueous‐based lithium recovery techniques have been developed and applied industrially (Figure 1c).^[^ ^19^, ^20^ ^]^ The variations of species in feed solutions directly impact the difficulty and efficiency of lithium recovery from water resources. Although traditional methods are well‐established, there exist critical technical and cost challenges. For example, while energy‐efficient and simple to operate, adsorption approaches suffer from the drawbacks of time‐consumption and high associated costs.^[^ ^21^ ^]^ Solvent extraction usually generates large amounts of toxic waste substances.^[^ ^22^ ^]^ Precipitation typically has low recovery rate and requires substantial time.^[^ ^23^ ^]^ Though these techniques have high technology readiness levels (TRLs) (**Figure** **2**), they have critical technical, environmental, and energy issues. Therefore, the development of techniques that have exceptional lithium recovery capabilities, low energy consumption, and high sustainability is desirable, in which membrane processes are considered a promising candidate.

Membrane separation process has been widely applied to gas, liquid, and ion conditions, with great contribution made to energy, healthcare, and chemical production industries.^[^ ^25^, ^26^, ^27^, ^28^, ^29^, ^30^ ^]^ Especially in recent years, with the rapid development of desalination, biomedicine, and ESS fields, ionic separation membrane technology has garnered significant attention.^[^ ^31^, ^32^, ^33^ ^]^ The membrane separation process offers advantages over traditional methods by eliminating the need for costly and environmentally harmful solvents. This process can achieve the increased separation efficiency under conditions that are both simple and continuous in operation, significantly reducing the need for extensive postseparation processing.^[^ ^34^, ^35^, ^36^ ^]^ Figure 2 illustrates the state‐of‐the‐art membrane‐based techniques for lithium extraction. In general, membranes in the recovery process can be divided into those that selective membranes via permeation and adsorption processes, and nonselective membranes serving as the support. The nonselective membranes are generally designed according to the requirements of specific applications.

While membrane processes in lithium recovery have received much research interest, as indicated by a marked surge in review publications,^[^ ^14^, ^35^, ^37^, ^38^, ^39^ ^]^ limited efforts have been made to understand the fundamentals of lithium transport in order to provide membrane design principles. Since the design requirements of nonselective membranes need to be adapted to the corresponding processes, this review will primarily focus on materials and structural aspects of selective membranes and aim to provide guidance for future research by discussing the working principles and latest research achievements.

## 2 Theories and Fundamentals of Membrane‐Based Lithium Recovery

### 2.1 Membrane Process for Single‐Species Selectivity

**Figure 3:**
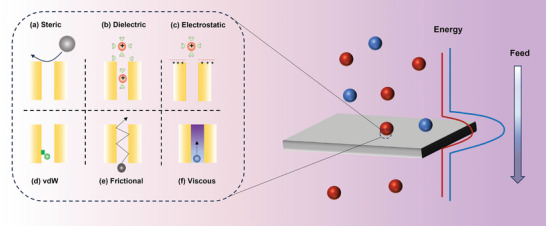
Illustration of membrane separation mechanisms. The red spheres stand for the substance allowed to transfer; the blue spheres are the rejected substances because of higher energy barrier.

Membrane, usually defined as an interphase, isolates two phases and restricts the transport of substances in a specific manner.^[^ ^36^ ^]^ Fundamentally, membrane‐based separation arises from the differences in transmembrane transport of substances.^[^ ^42^ ^]^ In describing the diffusion of a substance in a medium, Eyring's transition state theory (TST) is one of the widely accepted theoretical models.^[^ ^43^, ^44^ ^]^ According to TST, the diffusion of substances in a medium involves breaking any stabilizing molecular interactions found in the original environment and generating new molecular interactions within the medium of the forward direction. This process requires crossing unstable high potential energy states, which are also known as energy barriers.^[^ ^45^ ^]^ For transmembrane transport of substances, the energy barrier provided by the membrane is normally much larger than in pure media. Target species are anticipated to permeate through the membrane with low activation energies while other species should be rejected by the membrane due to high energy barriers. This general process usually involves a complex combination of steric hindrance,^[^ ^46^, ^47^ ^]^ electrostatic repulsion,^[^ ^48^, ^49^ ^]^ dielectric effect,^[^ ^50^ ^]^ van der Waals forces,^[^ ^51^ ^]^ intrapore friction,^[^ ^52^ ^]^ and viscous effect^[^ ^53^ ^]^ (**Figure** **3**). Steric hindrance can help sieve substances based on their size. For charged substances, they can be attracted or repelled by the charged membrane and produce different electrostatic effects depending on the charge differences. When charged ions are in an aqueous environment, the electric field attracts water molecules forming hydration shells. The hydrated ion dehydrates as it enters a smaller pore, creating a split energy barrier. In addition, the solute requires overcoming frictional and viscous interactions in the internal channels of the membrane, which are caused by physical collisions or chemical affinities. These separation mechanisms work together to tune the energy barriers for different species. Therefore, the flexible and precise adjustment of these factors is the key to achieving optimal separation performance under given conditions.^[^ ^42^, ^54^ ^]^

The well‐known solution‐diffusion model, which is used in many fields to describe mass transfer across membranes, can also be applied to ion transport.^[^ ^55^ ^]^ However, this model is usually used to describe nonporous homogeneous membranes and the theoretical performance deviates from the actual results when describing porous membranes.^[^ ^56^, ^57^ ^]^ The combination of Donnan steric pore model and dielectric expulsion mechanism takes into account the effects of dielectric repulsion, Donnan repulsion, and size sieving to provide important understanding of ion transport mechanisms and guidance for future membrane improvement.^[^ ^57^ ^]^

In addition to the membrane itself, other external factors such as driving forces and pH can influence the membrane separation process.^[^ ^58^ ^]^ For example, driving forces such as pressure, concentration, potential, and temperature difference provide the necessary energy to transport substances across membranes.^[^ ^59^, ^60^, ^61^ ^]^ Apart from their direct impact on energy consumption, different driving forces can also affect the separation efficiency and selectivity of the membrane, which needs to be considered during the separation process.

### 2.2 Theoretical Foundations and Design Principles of Lithium Selective Membrane

**Figure 4:**
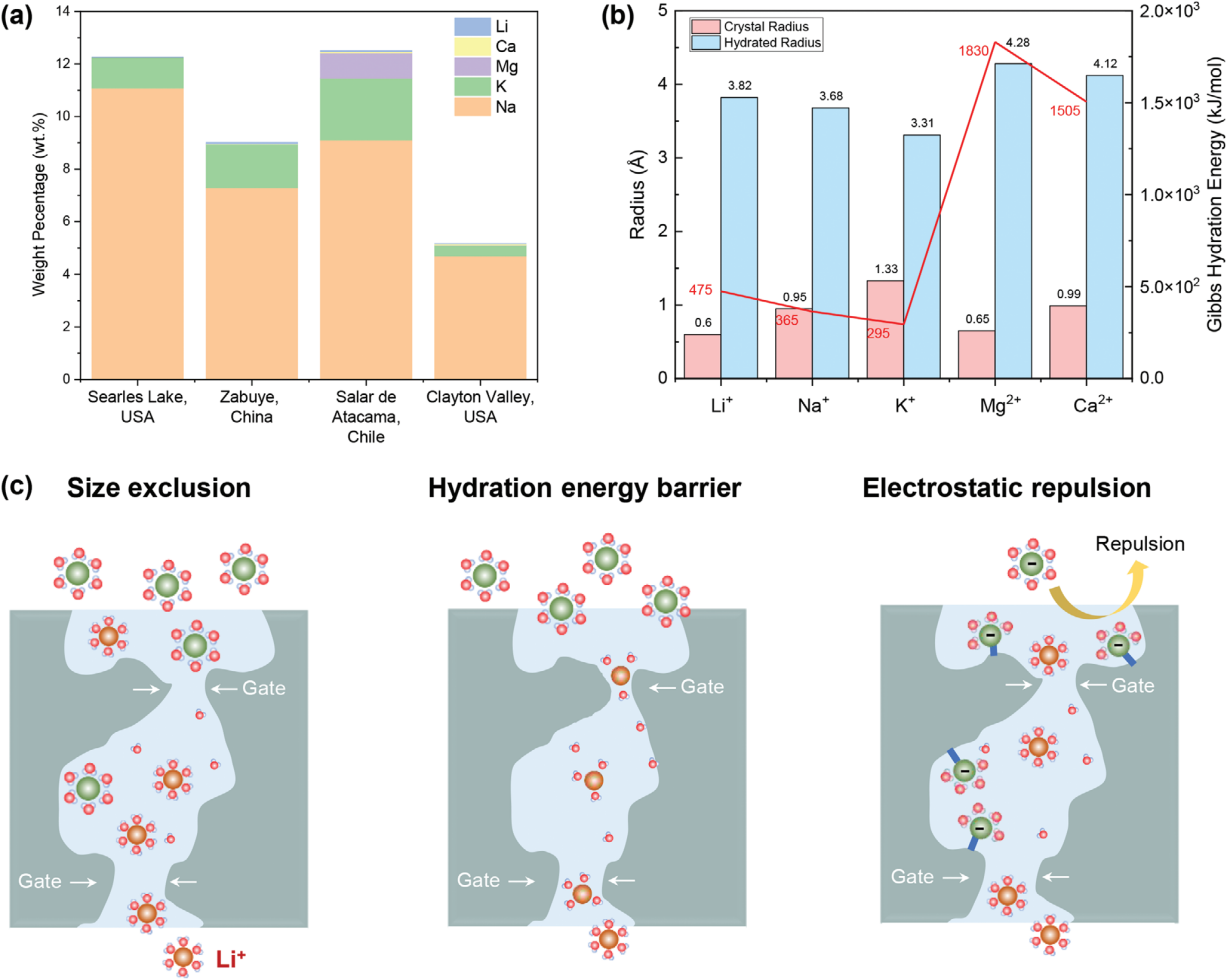
Working mechanisms for selectively transport Li^+^ ions. a) Element distribution in water resources. b) Ionic volume, hydrated volume, and hydration energy of Li^+^ and other coexisting ions. c) Lithium separation mechanisms.

Membrane processes for ion separation have undergone rapid development where techniques represented by nanofiltration (NF) membranes and ion exchange membranes (IEMs) have made remarkable progress. While considerable success has been achieved in the separation of ions with opposing charges, the separation of ions with the same charge, especially those with similar size and valence, still remains a challenge and is an active area of research.^[^ ^62^ ^]^ For instance, in addition to Li^+^,^[^ ^63^ ^]^ abundant coexisting cations such as Na^+^, K^+^, Ca^2+^, and Mg^2+^ are present in natural water resources (**Figure** **4**a). For the recycling of lithium from spent batteries, the coexistence of transition metal ions, e.g., Mn^2+^, Ni^2+^, and Fe^2+^, also needs to be considered. These cations are difficult to separate through traditional IEM techniques. Although NF membranes demonstrate separation ability toward certain species, it is still not ideal for separating (sub‐)nanoscale species, such as metal ions. How to effectively separate Li^+^ ions from these coexisting ions is therefore the main challenge in the recovery process.

Generally, membranes with lithium‐ion recovery ability can be designed from two principles: size exclusion effects and ion–membrane interactions. Separation through size exclusion typically involves the dehydration of metal ions. The ionic volumes and hydration volume/energy characteristics of Li^+^ with common coexisting cations in brines are shown in Figure 4b. Metal cations naturally form a hydration shell that is surrounded by water molecules in an aqueous environment due to electrostatic forces.^[^ ^64^ ^]^ Hydrated ions need to overcome certain energy barriers to (partially) dehydrate when they get engaged in channels smaller than the hydration size dimension^[^ ^64^ ^]^ (Figure 4c). Although hydrated lithium ions have slightly higher hydration energy and hydration size among alkali monovalent ions, their significantly lower hydration energy difference compared to hydrated multivalent ions like Mg^2^⁺ and Ca^2^⁺ provides favorable conditions for the separation of monovalent and multivalent ions. Notably, dehydrated lithium ions were shown to possess the fastest transport velocity of the alkali metal cations, which also contributes to the selective separation of Li^+^ ions.^[^ ^54^ ^]^ However, achieving dehydration while allowing the permeation of dehydrated Li^+^ ions presents a challenge for constructing membranes with suitabl size and well‐defined channels of sub‐nanometer diameter. Current research focuses on the development of microporous materials with angstrom pore sizes. For example, crown ether (CE), crystalline 9 metal–organic frameworks (MOFs), or some 2D materials with defined apertures have been demonstrated to be effective in lithium recovery.^[^ ^65^ ^]^

In addition to size exclusion effects, employing electrostatic repulsion, namely the Donnan effect, is another approach to achieving effective separation^[^ ^66^ ^]^ (Figure 4c). Incorporating charged units into membranes can repel undesired species with similar charges or those with high charge densities. Ions with higher charge are subjected to stronger electrostatic interaction in an electric field and vice versa. Multivalent cations, compared to Li^+^ ion, undertake stronger electrostatic repulsion from membranes with the same charge, enabling the differential transport rate of monovalent/multivalent ions. However, electrical forces from the charged membranes can reduce the ion permeability and lead to a decrease in recovery efficiency, e.g., anion‐exchange membranes and target cations, which requires to balance the trade‐off between selectivity and permeability during the lithium recovery process. It is worth mentioning that grafting charged functional groups within the membrane structure, especially in the channel, is considered to not only stabilize dehydrated ions, but also provide sites for the migration of ions, although the contribution of this part is still unclear and requires further investigation.^[^ ^49^ ^]^ Another type of ion–membrane interaction is the dielectric effect caused by differences in dielectric constants. However, although there are several reports regarding improving the selectivity of monovalent ions by tuning membrane hydrophobicity, we believe that this approach may not hold significant potential for commercial viability, because this approach may greatly sacrifice ion permeability.

According to these working principles, strategies to extract Li^+^ ions from a mixture of either multivalent or monovalent ions can be therefore proposed as follows.

1. Design the pore size to maximize the selectivity of Li^+^ ions toward other species. The pore channels within membranes can be adjusted on the sub‐nanoscale, which affords (partial) dehydration of Li^+^ ions so as to increase the permeation rate and enhance selectivity.
2. Enhancing the charge density of the surface and bulk of the membrane by functionalizing with functional/polar units.
3. Combining these strategies to modify sub‐nanometer channel walls with charged units to provide size exclusion and electrostatic repulsion effects.

It is important to note that in addition to the elucidated separation mechanisms, phenomena such as concentration polarization also needs to be taken into account, given its significant influence on the separation process. Typically, concentration polarization is undesirable for separation processes based on pressure, voltage, or solute self‐diffusion. In addition to reduced separation performance, it also results in high energy losses. Hence, the need to implement measures to mitigate its adverse effects.^[^ ^67^ ^]^

## 3 Evaluation Metrics

The number of publications on lithium recovery membranes has increased dramatically in recent years, and effective evaluation of the performance of various innovative membranes, such as adsorption capacity, permeability, and stability, is crucial. The establishment of an effective evaluation metrics system for the evaluation and comparison of lithium recovery membranes is recommended. Notably, in many recent related studies, alongside the widely concerned key metrics such as recovery efficiency and selectivity, performance such as lifespan and energy consumption are also garnering increasing attention, which are of great significance for practical application.

It should be noted that many studies employ various methods and systems for the evaluation of the lithium extraction performance of membranes, lacking a unified standard. For instance, multi‐ion systems are evidently more representative of practical applications compared to single‐ion systems, as ion competition is believed to have a direct impact on ion transport and separation. Additionally, the application of an electric field significantly influences separation performance, particularly for the test with only ion conductivity, which apparently has great limitations for assessing ion separation capability. Therefore, metrics evaluation needs to be analyzed in conjunction with specific operational systems.

### 3.1 Extraction Efficiency and Selectivity

As noted, while Li is widely distributed in diverse water resources, the effective extraction of Li^+^ remains a significant challenge due to its relatively low concentration and the presence of coexisting ions. A successful membrane‐based recovery process necessitates a high degree of Li^+^ selectivity while rejecting other solutes. Without high selectivity, inadequate purity may lead to impurities precipitating during subsequent precipitation process, rendering the obtained Li compound unusable. In addition to selectivity, a sufficiently high Li extraction efficiency is imperative for industrial production. It is noteworthy that the concentration of Li^+^ and types of coexisting ions in various lithium sources, even from within the regional deposits, exhibit considerable variation, which has a direct impact on the separation effect of the membrane.

Recently, many membranes exhibiting excellent lithium‐ion selectivity have been reported, as evidenced through various experimental test methods. It should be noted that membrane‐based lithium recovery technologies are still at an early stage and considerable research efforts are being devoted to develop effective protocols. Many research test methods are based on different TRLs (e.g., electrodialysis, current–voltage (*I*–*V*), adsorption/desorption, and pressure/concentration‐driven diffusion) with varied testing systems (e.g., single‐ion solution, binary ion solution, and real/artificial lithium resource), which require reasonable deductions and conclusions from the reported results. Different methods and parameter conditions can significantly impact the results. For example, single‐salt solutions cannot account for the competition effect of ions, and low lithium‐ion ratios in real salt water typically face higher separation difficulties. The test with real lithium sources or artificial nonequivalent mixed solutions with higher TRLs is usually closer to the actual industrial application environment and binary mixed solutions can also reflect the separation ability of specific ion pairs.

Ion recovery is used to describe the ability of a selective membrane to recover target ions. For the recovery of Li^+^, the expression is usually the proportion of Li^+^ extracted relative to the total lithium ions in the original solution. The recovery rate is generally calculated according to Equation (1).

(1) $$\eta =\frac{m_{r,\;Li^{+}}}{m_{r,\;Li^{+},t=0}}$$

where $m_{r,\;Li^{+}}$ denotes the mass of recovered Li^+^ ions and $m_{r,\;Li^{+},t=0}$ denotes the mass of Li^+^ ions in the feed water.

Typically, membrane‐based recovery with lithium selectivity can be performed by both adsorption and permeation processes for Li extraction. Either way, membranes with excellent Li extraction efficiencies are usually characterized by a higher Li recovery capacity per unit mass/area of membrane in a shorter period of time, which can therefore be calculated from the ion flux *J* (Equation (2)) or the adsorption capacity *Q* (Equation (3))

(2) $$J_{t}=\frac{\left(C_{t}-C_{0}\right)V}{A\Delta t}\;$$

(3) $$Q_{\mathrm{Li}}=\frac{\left(C_{0}-C_{e}\right)V}{m}\;$$

where *C*_0_ and *C_t_* (mg L^−1^) are the initial concentration of Li^+^ in the recovered solution and the concentration at time *t*; *V* (mL) is the volume of the recovered solution; *A* (cm^2^) is the contact area; *d* is the thickness of the membrane; *C*_e_ (mg L^−1^) and *m* (mg) are the equilibrium concentration of Li^+^ and the mass of the membrane in the contact area, respectively.

Since the adsorption process is discontinuous, repeated adsorption–desorption processes are required. Therefore, the kinetics of the adsorption process can also be expressed by Equation (4)

(4) $$Q_{t}=\frac{\left(C_{0}-C_{t}\right)V}{m}\;$$

where *Q_t_* (mg g^−1^) and *C_t_* (mg L^−1^) are the adsorption capacity and concentration of Li^+^ at different times *t*, respectively.

Selectivity can be expressed as the ratio of the concentration of the recovered target ion (i.e., Li^+^) to the concentration of other ions (Equation (5))

(5) $$\eta =\frac{C_{r,Li^{+}}}{C_{r,Li^{+}}+\;C_{r,i}}\;i=Na^{+},\;K^{+},\;Mg^{2+},\;Ca^{2+}$$

where $C_{r,\;Li^{+}}$ (mg L^−1^) is the concentration of Li^+^ recovered and *C*_r, i_ is the concentration of other ions recovered. In addition, selectivity can be also expressed as the ratio of the adsorbed capacity or flux of Li^+^ to other ions (Equations (6) and (7))

(6) $$\alpha =\frac{Q_{Li^{+}}}{Q_{i}}\;$$

(7) $$\begin{array}{c} S=\frac{J_{Li^{+}}}{J_{i}}\;\\ \;i=Na^{+},K^{+},Mg^{2+},Ca^{2+},\mathrm{etc}. \end{array}$$

where $Q_{Li^{+}}$ and $J_{Li^{+}}$ are the adsorption capacity and flux of Li^+^, respectively, and *Q*_i_ and *J*_i_ are the adsorption capacity and flux of other coexisting ions. A detailed table (**Table** **1**) for comparing the selectivity of different cutting‐edge materials is provided in Section 5.

**Table 1:** Summary of materials and methods used for lithium separation.

Material		Lithium extraction mechanism	Selectivity (equimolar mix solution)^a^ ^)^	Disadvantages	Refs.
Crown ether	12‐crown‐4 18‐crown‐6 15‐crown‐5	Size exclusion; chelate effect	*α* (K^+^, Na^+^, Mg^2+^): 2–50	Discontinuity; poor cycle stability; low adsorption capacity	[4, 6, 76, 77, 78, 79, 80, 81, 85, 156]
Ceramics	L(H)MO; L(H)TO; LICGC	Size exclusion (knock‐in/knock‐off mechanism)	*α* (K^+^, Na^+^, Mg^2+^): 50–1000	Poor membrane mechanical property; long recovery time; high cost; chemical stability in acidic solutions	[88, 89, 90, 95, 96, 97, 98, 99, 100]
MOFs	ZIF‐8; HKUST‐1; UiO‐66	Size exclusion	*α* (K^+^, Na^+^, Mg^2+^): 1–10	Limited monovalent ions separation; stability issues in harsh solutions; high cost	[113, 114, 115, 116, 117, 119, 120, 121, 123]
2D materials	Graphene; MXene;	Size exclusion	*α* (K^+^, Na^+^, Mg^2+^): 1–20	Limited monovalent ions separation; high cost; poor stability; complex membrane formation	[120, 121, 122, 123]
Positively charged polymers	Amine‐based; guanidine; thiol	Electrostatic effect	*α* (Mg^2+^): 10–100	Limited monovalent ions separation; decreased permeability	[138, 140, 141, 143, 144, 145, 146, 150, 152, 153, 154, 155]

^a)^ *α* stands for the selectivity of the concentration of Li ions toward those of other salts.

The effect of ion concentration in the feed solution is taken into account, e.g., coexisting ions in the recovery solution above the lithium ion concentration that may result from other metal ions in the brine well above the lithium ion concentration. The concentration of solutions can be further normalized to more accurately express the selectivity of the membrane for different ions (Equations (8) and (9))

(8) $$\alpha =\frac{Q_{\mathrm{Li}+}C_{i}}{Q_{i}C_{\mathrm{Li}+}}\;$$

(9) $$S=\frac{J_{\mathrm{Li}+C_{i}}}{J_{i}C_{\mathrm{Li}+}}\;$$

*C*_Li +_  and *C*_i_ (mg L^−1^) represent the equilibrium concentrations of Li^+^ and other ions in solution, respectively.

The migration of ions in aqueous environments, especially at the nanoscale within membranes, is a complex process. Phenomena such as competition or concentration polarization caused by coexisting ions can significantly affect separation outcomes. Additionally, lithium recovery efficiency is influenced by factors such as driving force, pH value, and temperature. For example, applying pressure or an electric field can enhance ion permeation efficiency but may compromise selectivity. There exists a trade‐off between selectivity and permeability: higher permeability typically results in lower selectivity and vice versa. To properly regulate this balance, it is crucial to design rigid and more stable membrane structures, which are key to optimizing both selectivity and permeability.

**Figure 5:**
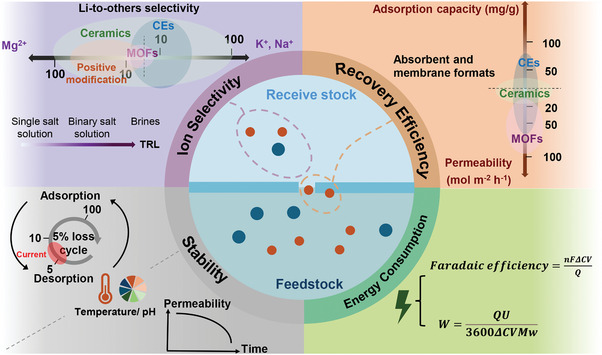
Evaluation metrics for the lithium selective membranes include recovery efficiency, selectivity, stability, energy consumption. CEs: crown ethers. MOFs: metal–organic frameworks. Positive modification means the molecular structure is functionalized with positively charged functional groups.

At present, some existing materials have shown excellent lithium recovery capabilities. For instance, CEs and lithium‐based ceramic materials, with their very suitable cavity sizes, can be used for selective adsorption. These materials demonstrate outstanding selectivity for both monovalent and multivalent ions, in which certain ceramic even shows selectivity coefficients ranging from 100 to 1000. MOFs with relatively large surface areas exhibit high lithium ion permeability (10–90 mol m^−^^2^ h^−^¹) in both powder and membrane forms. Additionally, positively charged modified polymer membranes show very stable and exceptional separation capabilities for divalent ions, with selectivity coefficients between 10 and 100 (**Figure** **5**).

### 3.2 Stability and Lifespan

Li‐rich resources typically have complex and harsh environments. Factors including temperature, pH, and ion concentration as well as oxidation/reduction of certain ions (e.g., V^3+^/V^4+^) directly influence membrane stability, thereby affecting their longevity and recovery efficiency. In addition, during the battery recycling process, the hydrometallurgical method usually has a much harsher condition, which will pose a greater challenge to the stability of the membrane than compared to natural water sources, posing greater challenges to membrane stability.^[^ ^68^ ^]^ Although there are few reports specifically on lithium recovery membranes, similar studies have shown significant stability challenges in complex environments, such as aqueous flow batteries.^[^ ^27^, ^69^ ^]^ Effective next‐generation membranes should possess stable molecular structures that resist concentrated H+ and OH− (e.g., strongly fluorinated modified hydrocarbon^[^ ^70^ ^]^), exhibit limited swelling in acids or bases, provide significant cycling stability, and show minimal fouling issues.

In the adsorption recovery process, multiple adsorption/desorption cycles can damage binding sites within the membrane and disrupt its physical structure, leading to a degradation of mechanical properties. For example, Li extraction adsorbents/membranes generally experience a 5% loss of adsorption over 5–10 cycles (red area of the Stability section in Figure 5). We anticipate that future materials can maintain high stability (e.g., 5% loss over 100 cycles) by tuning the structures to mitigate material fouling and enhance thermal, chemical, and electrochemical stability. Additionally, contamination and blockage pose significant challenges for permeation membranes, greatly decreasing the permeability and selectivity.

Given the scenarios in commercial production, membranes are required to operate without performance degradation over many years. In fact, issues raised from contamination have already received attention. To enhance the membrane stability, inorganic materials that are highly hydrophilic and antimicrobial are introduced, but this increases the capital cost. In particular, membranes with complex structures and synthesized by sophisticated processes face challenges in their stability performance, despite their improved ion recovery. In addition, the time and frequency in many reports are still short for evaluation of industrial performance and proper evaluation for Li recovery over time is very necessary to develop this field.

### 3.3 Energy Consumption and Cost

In addition to separation performance, energy and costs, both for manufacture and usage, are gaining more attention. Typically, the driving forces, such as the applied external pressure, is one of the primary sources for energy consumption. Specifically, employing external pressure can significantly drive the permeation of target ions and compensate the disadvantages of low self‐diffusion rates for ions. Additional energy consumption caused by continuous processes should also be considered.

For separation processes based on electrical drive, the energy consumption can also be expressed in terms of the Faraday efficiency (Equations (10) and (11))

(10) $$\mathrm{Faradaic}\;\mathrm{efficiency}\;=\frac{nF\Delta CV}{Q}\;$$

(11) $$W=\frac{QU}{3600\Delta CVMw}\;$$

where *n* is the charge number of ions, *F* is the Faraday constant, Δ*C* represents the concentration variation of ions, *V* is the anolyte volume, and *U* is the applied voltage.

Although material costs are not frequently mentioned in many reports, they are crucial for industrial large‐scale production applications. For example, some lithium ceramic materials, despite their outstanding lithium recovery capabilities, are costly to produce. By contrast, materials with slightly lower selectivity, such as MOFs, have achieved large‐scale production and are closer to practical application. Additionally, some modifications based on NF and electrodialysis (ED) membranes also exhibit high TRL and economic viability. Therefore, material cost is a factor that cannot be ignored in the integration of industry, academia, and research.

## 4 Pore Size Exclusion

Given the smallest ionic size of Li^+^ and the significant hydration energy difference with multivalent ions, adjusting the appropriate pore size to separate Li^+^ from coexisting ions by ion‐size exclusion via dehydration is considered to be the most effective method. As constructing selective channels at an angstrom scale is highly challenging, cutting‐edge microporous materials, Li^+^‐conducting ceramics, and polymers provide viable solutions.^[^ ^71^, ^72^ ^]^

### 4.1 Crown Ether

CEs are a class of heterocyclic organic compounds that contain multiple ether groups.^[^ ^73^ ^]^ Due to ion–dipole interactions, host–guest complexes exhibit superb selectivity with certain cations, particularly the alkali metal ions.

**Figure 6:**
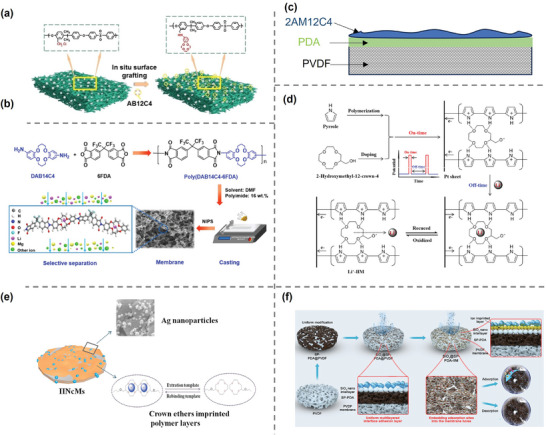
Lithium‐recovery based on crown‐ether chemistries. a) Preparation of polysulfone surface graft 4′‐aminobenzo‐12‐crown‐4‐ether membrane. Reproduced with permission.^[^ ^75^ ^]^ Copyright 2020, PNAS. b) Schematic of the preparation and the structure of Poly(DAB14C4─6FDA)‐based membrane. Reproduced with permission.^[^ ^76^ ^]^ Copyright 2020, Elsevier. c) Illustration of 2‐(allyloxy) methyl‐12‐crown‐4‐based IIP layer on PVDF substrate, where PDA serves as an interlayer. d) Schematic of the synthesis and recovery process through electrochemically switched ion exchange (ESIM) method. Reproduced with permission.^[^ ^77^ ^]^ Copyright 2020, Elsevier. e) Ag‐modified 2AM12C4‐based membrane with improved hydrophilicity and performance. Reproduced with permission.^[^ ^78^ ^]^ Copyright 2018, Elsevier. f) Schematic illustration of synthesis and adsorption process of SiO_2_@SP─PDA─IIM. Reproduced with permission.^[^ ^4^ ^]^ Copyright 2022, Elsevier.

Among CE families, 12‐crown‐4 (12C4) and its analogs (such as 2‐hydroxymethyl‐12‐crown‐4, benzo‐12‐crown‐4, etc.) have received particular attention. Due to the appropriate macrocyclic cavity size (0.72–0.81 Å) and chelate effect of ether groups, these exhibit excellent selective binding ability for Li^+^ ions of similar size, effectively screening out larger Na^+^, K^+^, and Ca^2+^ ions. Although Mg^2+^ and Li^+^ are similar in volume, the slight differences still affect their binding within the CE.^[^ ^74^ ^]^ Li et al.^[^ ^75^ ^]^ grafted aminobenzo‐12‐crown‐4 onto chloromethylated polysulfone via in situ surface grafting. The maximum adsorption capacity of prepared membrane was 51.99 mg g^−1^ in a 700 mg L^−1^ Li^+^ solution and the separation coefficients were reached 38.43, 33, 27, and 3.4 in the equimolar mixed solution for K^+^, Na^+^, Ca^2+^, and Mg^2+^, respectively (**Figure** **6**a). Instead of side chain, Zhu et al.^[^ ^76^ ^]^ synthesized a novel dibenzo‐14‐crown‐4 ether‐based polyimide (Poly(DAB14C4─6FDA)) through step growth polymerization between di(aminobenzo)‐14‐crown‐4 and 4,4′‐(hexafluoro‐isopropylidene) diphthalic anhydride (6FDA) (Figure 6b). The copolymer was fabricated into porous self‐standing membrane through nonsolvent‐induced phase separation. The membrane exhibited a lithium‐ion adsorption capacity of 34.05 mg g^−1^ (Li^+^: 500 mg L^−1^) and Li^+^/Mg^2+^ selectivity of 23.5 in the mixture solution. After five cycles of testing, the adsorption capacity decreased by only 2.3%.

Chemical bonding of crown ethers to polymer backbone or side chains is not an easy task, especially considering the mechanical stability, porosity, and hydrophilic/hydrophobicity of the modified polymers after membrane formation. To address these issues, some researchers have adopted a simpler and more controllable design of composite membranes. This kind of structure involves introducing a substrate and performing in situ synthesis or attaching functional layers on the surface, thereby enhancing mechanical performance while obtaining specific properties. Additionally, this asymmetric structure sometimes and effectively improves the balance between selectivity and permeability of substances. For example, ion‐imprinted membrane (IIM) combines ion‐imprinted polymer (IIP) and membrane separation technology to retain the advantages of membrane processes while achieving high ion selectivity.^[^ ^5^ ^]^ In this case, CEs serve as the ligands with Li^+^ templates and cross‐linking monomers to synthesize network polymers. Cavities with Li^+^ specific dimensions will be retained after the Li^+^ template is removed, resulting in excellent selectivity and recombination capability.^[^ ^5^ ^]^ Sun et al.^[^ ^79^ ^]^ fabricated an ion‐imprinted layer (IIL) synthesized from 2‐(allyloxy)methyl‐12‐crown‐4 on a polyvinylidene fluoride (PVDF) substrate, with polydopamine (PDA) introduced as an adhesive intermediate layer (Figure 6c). The introduction of CE showed an enhanced Li^+^ binding capacity (27.1 mg g^−1^) in a 200 mg L^−1^ LiCl solution and selectivity factor of 4.42 for Li^+^/Mg^2+^. Liu et al.^[^ ^77^ ^]^ synthesized Li^+^‐ion‐imprinted membranes through unipolar pulsed electropolymerization using pyrrole as a conductive and cross‐linking agent and 2‐hydroxymethyl‐12‐crown‐4 ether as the trapping agent. To avoid the problem of void destruction caused by the traditional acid leaching process, electrochemically switched ion exchange was employed to adsorb and regenerate Li^+^ in solution (Figure 6d). The IIM showed a Li^+^ adsorption capacity of 16.4 mg L^−1^ in the 10 mg L^−1^ Li^+^ solution at −0.2 V adsorption potential but retained 95.88% of its initial adsorption capacity after 5 cycles.

It is important to note that for adsorption/desorption processes, cyclic stability is one of the most essential metrics for industrial applications, especially in complex lithium‐rich aqueous environments, which require membranes to be fouling‐resistant and structurally stable for a sufficient service life.^[^ ^82^ ^]^ Certain hydrophilic inorganic materials have demonstrated great antifouling properties.^[^ ^83^, ^84^ ^]^ Sun et al.^[^ ^78^ ^]^ introduced highly hydrophilic and antibacterial Ag nanoparticles into the PDA layer of the 2‐(allyloxy)methyl‐12‐crown‐4 (2AM12C4)‐based IIM (Figure 6e). The modified membrane showed effectively enhanced permeation selectivity and regeneration stability, which is likely due to the hydrophilicity and fouling resistance of Ag particles. Similar improvements were obtained in the modification of 12C4‐based IIMs with TiO_2_ and SiO_2_, respectively.^[^ ^6^, ^85^ ^]^ However, this method places high demands on the uniformity of the PDA bonding layer and nanoparticle dispersion, as nonuniform intermediate layers can not only affect the modification effectiveness of inorganic particles but also decrease the performance of the IIL. He et al.^[^ ^4^ ^]^ synthesized a novel multilayer interlayer (SiO_2_@SP─PDA) by the oxidation of sodium periodate (SP) and introduced SiO_2_ under slightly acidic conditions (pH = 5) (Figure 6f). The adsorbent layer was constructed on PVDF and had better homogeneity, which could prevent agglomeration during the grafting process. The lithium adsorption capacity was increased to 231.77 mg g^−1^ (Li^+^: 500 mg L^−1^) with Li^+^ selectivity of 4.36, 5.03, and 3.55 for K^+^, Na^+^, and Mg^2+^. Incorporating CE structure into membrane has proven to be a feasible strategy, with excellent lithium adsorption capacity and selectivity obtained. In general, a higher density of CE tends to result in more efficient lithium adsorption. Additionally, the hydrophilicity of the membrane directly influences its recovery performance. Although the tested number of cycles is limited, it can reflect the objective stability of the membrane to some extent, which, however, may face more severe challenges in the actual recovery solution.

**Figure 7:**
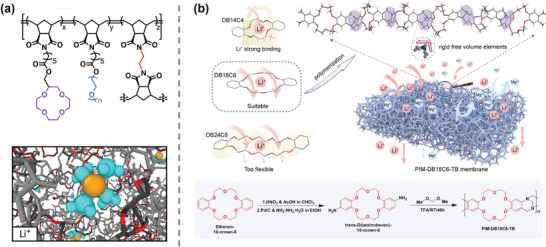
Polymer‐backbone‐implanted crown‐ether membrane. a) Chemical structure of copolymer (left) and molecular dynamics (MD) renderings of 12C4‐based membranes. Reproduced with permission.^[^ ^80^ ^]^ Copyright 2020, PNAS. b) Illustration of PIM─DB18C6─TB and schematic of the synthetic routes. Reproduced with permission.^[^ ^81^ ^]^ Copyright 2023, Elsevier.

While such surface‐modified composite membranes exhibit good Li^+^ selectivity and recovery ability, the relatively complex membrane structure is difficult to prepare and leads to poor stability in use, posing significant constraints on their commercial large‐scale production. Warnock et al.^[^ ^80^ ^]^ introduced 12C4 into polymer side chains through ring‐opening copolymerization, while also incorporating poly(ethylene oxide) side chains and a cross‐linker to enhance the hydrophilicity and mechanical properties of the polymer (**Figure** **7**a). The prepared membranes exhibited a permeability selectivity as high as 2.3 in equimolar Li^+^/Na^+^ single‐salt solutions, but decreased to 1.1 in mixed solutions, which is likely due to the differences in electrostatic interactions between the metal cations and anions. Notably, instead of employing 12C4, Dong et al.^[^ ^81^ ^]^ incorporated dibenzo‐18‐crown ether‐6 (DB18C6) with a slightly larger cavity into the polymer backbone via Tröger's base (TB) polymerization. DB18C6 was chosen because of its relatively rigid backbone and moderate binding capacity, allowing for the selective permeation of Li^+^ based on the differences in binding affinity with different ions (Figure 7b). The experimental results showed a Li^+^/Mg^2+^ selectivity of 35.8 from a double ion diffusion test and 24.4 for electrodialysis test, respectively. This method not only solves the problem related to the complexity and instability of multilayer structured membranes but also effectively addressed the discontinuity issue caused by the adsorption process, thereby improving the recovery efficiency. It is worth mentioning that, in addition to the CE species, the rigidity of TB is crucial as it prevents the polymers from packing efficiently, creating free channels for ion transport. This polymer of intrinsic microporosity (PIM) has already exhibited excellent performance in gas separations and shows great potential in the field of ion separation.^[69,86,]^ The material is microporous and does not possess a network of covalent bonds, hence it is highly soluble, allowing it to form membranes, making it very promising for future ion separation research.

CE‐based membranes exhibit impressive recovery capabilities and excellent separation efficiencies for both monovalent and multivalent ions. The structure of these membrane significantly influences their performance. For example, composite membranes incorporating 12C4 offer a more facile strategy, however, they have reduced selective adsorption capacity compared to freestanding membranes. Besides, the study of selective permeability of CE‐based membranes is particularly valuable as these membranes maintain exceptional separation capabilities and offer a potential solution to the discontinuity observed in the adsorption process.

### 4.2 Li‐Containing Inorganic Membranes

**Figure 8:**
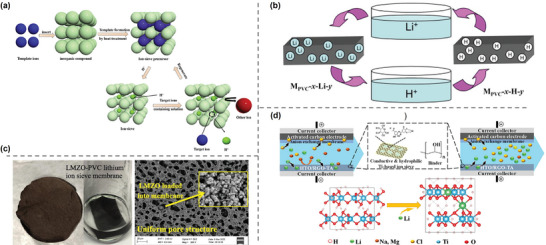
Lithium recovery with ceramic/polymer absorbent membranes. a) Schematic representation of the synthesis of ion sieves and work process. Reproduced with permission.^[^ ^87^ ^]^ Copyright 2020, Elsevier. b) Work process of LMO─PVC composite membrane. Reproduced with permission.^[^ ^88^ ^]^ Copyright 2014, Elsevier. c) Photos and scanning electro microscope (SEM) images of zirconium‐coated lithium ion sieve precursor (LMZO)─PVC lithium‐ion sieve membrane. Reproduced with permission.^[^ ^89^ ^]^ Copyright 2023, Elsevier. d) Illustration of HTO/rGO─TA(PVA) electrode and work process. Reproduced with permission.^[^ ^90^ ^]^ Copyright 2023, American Chemical Society.

Some inorganic materials also show an excellent ability of Li^+^ selective extraction.^[^ ^91^, ^92^, ^93^ ^]^ These type of materials, known as the lithium ion sieve (LIS), are considered to be a very promising method for lithium recovery due to its intrinsically Li^+^‐ion conducting structure.^[^ ^34^, ^94^ ^]^ The high selectivity of LIS mainly stems from the lithium‐specific sites retained after elution of Li^+^ ions from the presynthesized Li‐containing precursors. Because dehydrated Li^+^ has the smallest size among metal ions, only Li^+^ can diffuse through LIS, while rejecting large‐sized ions^[^ ^87^ ^]^ (**Figure** **8**a).

Widely studied Li^+^ adsorption inorganic materials include lithium manganese oxides (LMOs) and lithium titanium oxides in the format of powders, which are known for their exceptional lithium adsorption capacity, regeneration ability, and stability. However, the direct use of LIS powder as adsorbents leads to problems including high pressure drop, powder loss, and high energy consumption. Given these challenges, mixed matric membranes (MMMs) were developed by embedding LIS particles within a polymer matrix. Zhu et al.^[^ ^88^ ^]^ prepared a MMM based on polyvinyl chloride (PVC) with Li_1.6_Mn_1.6_O_4_ incorporated (Figure 8b). The membrane with the optimal composition exhibited an adsorption capacity of 26.64 mg g^−1^ in a 150 mg L^−1^ Li^+^ solution, achieving 86.5% of the capacity of the equivalent LIS powder, demonstrating the high adsorption capability retained of embedded LIS. Furthermore, the membrane shows excellent selectivity for Li^+^, which was more than an order of magnitude greater than that for the coexisting ions. Wang et al.^[^ ^89^ ^]^ prepared PVC‐based MMM with zirconium‐coated lithium ion sieve precursor LMZO, incorporating polymethyl methacrylate and polyvinylpyrrolidone (PVPk30) to improve hydrophilicity and porosity. The PVC─LMZO membranes exhibited a lithium adsorption capacity of 18.33 mg g^−1^ in a real brine with a Li^+^ concentration about 130 mg L^−1^ (Figure 8c). Zhang et al.^[^ ^90^ ^]^ prepared a membrane electrode based on H_2_TiO_3_ (HTO) ion sieves with the addition of reduced graphene oxide (rGO) and tannic acid (TA) to enhance the conductivity and surface hydrophilicity (Figure 8d). The adsorption capacity of the composite membrane was enhanced from 10.1 to 25.2 mg g^−1^ in a solution containing 303 mg L^−1^ Li^+^ after 2 h under electric field. These studies demonstrated that the blended LIS retained a high degree of adsorption capacity while benefiting from the support of the polymer matrix. Beyond the LIS content, the polymer matrix also influences the recovery properties, including mechanical strength, hydrophilicity, and porosity.

**Figure 9:**
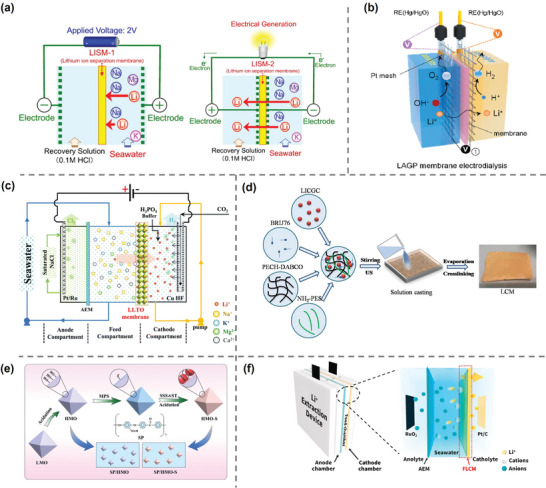
Electrodialysis using ceramic and polymer composites. a) Initial ED experiment (left) and improved method (right) by using Li^+^ sieving membrane. Reproduced with permission.^[^ ^95^ ^]^ Copyright 2014, Elsevier. b) LAGP membrane for ED experiment. Reproduced with permission.^[^ ^96^ ^]^ Copyright 2023, Elsevier. c) Illustration of three‐compartment electrical cell to continuously enrich lithium from feed solution. Reproduced with permission.^[^ ^97^ ^]^ Copyright 2021, Royal Society of Chemistry. d) Schematic of preparation of LICGC‐contained composite membrane. Reproduced with permission.^[^ ^98^ ^]^ Copyright 2020, Springer Nature. e) Synthesis process of hydrogen manganese oxide (HMO), HMO─S, and their hybrid membrane. Reproduced with permission.^[^ ^99^ ^]^ Copyright 2018, John Wiley and Sons. f) Illustration of the electrocatalytic‐driven Li^+^ extraction device. Reproduced with permission.^[^ ^101^ ^]^ Copyright 2023, Elsevier.

On the other hand, these ceramic inorganic materials have also demonstrated their potential and feasibility to be used in membrane dialysis processes, solving the problem of low recovery efficiencies in adsorption process limited by discontinuous property. Innovatively, Hoshino^[^ ^95^ ^]^ performed the first ED experiment for Li^+^ extraction from seawater using a lithium ion conductive glass‐ceramic (LICGC) membrane with high lithium conductivity. The positive and negative electrodes were attached on both sides of the membrane surface, aiming to solve the problem of low ion flux and poor stability in the common ED process (**Figure** **9**a). The membrane achieved a recovery rate of 7% after 72 h and exhibited extremely high Li^+^‐ion selectivity without applied voltage. Jiang et al.^[^ ^96^ ^]^ applied Li_1.5_Al_0.5_Ge_1.5_(PO_4_)_3_ (LAGP) ceramic membranes to extract 99.7% and 96.5% Li^+^ from 0.1 and 0.01 m LiOH feed solution, respectively, into 1 m LiOH receiving solution in a similar manner, although a sharp increase in resistance and energy consumption needed to be addressed at lower concentration (Figure 9b). Li et al.^[^ ^97^ ^]^ used Li_0.33_La_0.56_TiO_3_ (LLTO) membranes to enrich lithium from Red Sea seawater samples by 43 000 times under a continuous electrodriven process and successfully precipitated lithium phosphate with a purity of 99.94% from the enriched solution (Figure 9c). Innovatively, Xu et al.^[^ ^68^ ^]^ reported a “lithium‐rich electrode || Li_7_La_3_Zr_2_O_12_@bis(trifluoromethyl)sulfonyl)azanide and poly(3‐hexylthiophene‐2,5‐diyl)(LLZTO@LiTFSI + P3HT) || LiOH” system to achieve double‐sided and roll‐to‐roll recycling of electrodes. The LLZTO solid electrolyte membrane was modified with LiTFSI + P3HT to improve its stability in solution, thereby effectively enriching Li^+^ in materials such as LiFePO_4_. This system can produce hydrogen while obtaining high‐purity LiOH, providing excellent economic and environmental benefits. The excellent selectivity of the ceramic membrane confirms the feasibility of LIS for lithium permeation recovery processes and paves the way for further research. However, the fragile and unstable characteristics of inorganic membranes still retained, especially in alkaline media.

In order to solve the problem of poor mechanical stability of ceramic membranes, Ounissi et al.^[^ ^98^ ^]^ doped LICGC into an anion exchange polymer polyepichlorohydrin‐1,4‐diazabicyclo[2.2.2]octane(PECH─DABCO) copolymerized with amino–polyether sulfone (NH_2_─PES) (Figure 9d). In diffusion experiments, composite membranes with 50.5% LICGC content showed the highest Li^+^ flux of 30.53 × 10^−9^ mol cm^−2^ s^−1^ in a 0.05 m single‐salt solution and selectivity coefficients up to 376 (Li^+^/Na^+^), although there was a slight degradation of the performance after 72 h, which could be attributed to the gradual release of the LICGC ceramics due to the mechanical action of the polymer. Zhang et al.^[^ ^99^ ^]^ used sulfonated poly(ether ether ketone) as the polymer matrix and incorporated with acidified LMO (HMO) and its sulfonated derivative (S─HMO). Compared to HMO, S─HMO not only greatly improved Li^+^ flux and selectivity, but also exhibited better stability. This improvement can be attributed to the ionic conductivity of the sulfonic acid groups and their good compatibility with the matrix (Figure 9e). Similarly, Saif et al.^[^ ^100^ ^]^ prepared composite membranes by incorporating polystyrene sulfonate sodium salt (PSS─Na), lithium triflate, and HMO into a sulfonated polyether sulfone (SPES) matrix. The membranes exhibited Li^+^/Mg^2+^ binary separation factors of 9.1 and 5.0 in diffusion dialysis and electrodialysis tests, respectively. Shen et al.^[^ ^101^ ^]^ used sodium super‐ionic conductors (NASICON)‐type ceramics Li_1.3_Al_0.3_Ti_1.7_(PO_4_)_3_ as an ionic sieve and mixed in a 1:1 ratio in a poly(vinylidene fluoride‐co‐hexafluoropropylene) (PVDF─HFP) matrix to prepare membranes. The lithium extraction was carried out on dilute seawater samples by a homemade ED device (Figure 9f). After 5 h, the Li^+^ concentration in the receiving chamber was increased by 2.778 times compared to the original seawater and the Mg^2+^ and Ca^2+^ concentrations were reduced to 0.744% and 1.249% of the original concentrations.

Ceramic materials, used for adsorption or dialysis, demonstrate ultrahigh selectivity for coexisting ions, making them potential for lithium recovery from salt lakes or spent batteries. However, their relatively poor mechanical properties pose a challenge. The transport pathway of lithium ions in composite membranes is complex, influenced by polymers, fillers, and their interfaces. Higher ceramic content favors formation of transport networks, thus enhancing selective dialysis of target ions. However, excessive load can weaken membrane's mechanical properties or even cause failure, while too low a content results in poor performance due to the inability to form ion pathways.

### 4.3 Metal–Organic Frameworks as Components for Membranes

MOFs, consisting of metal ions or clusters coordinated with organic ligands, have received much attention in the field of storage,^[^ ^102^, ^103^, ^104^ ^]^ separation,^[^ ^105^, ^106^ ^]^ and sensing^[^ ^107^ ^]^ due to their sub‐nanometer pore size, high porosity, and excellent ion conductivity.^[^ ^108^ ^]^ Although many studies have demonstrated the excellent adsorption and separation capabilities of MOF materials for gases and some heavy metals, size‐based separation still poses challenges when dealing with Li^+^ and coexisting ions, which have smaller sizes.^[^ ^106^, ^109^ ^]^

**Figure 10:**
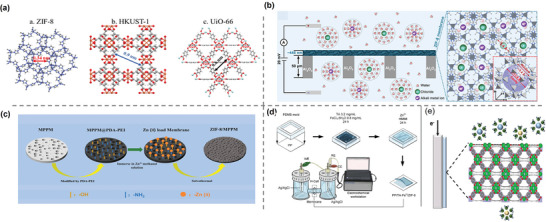
MOF and MOF‐based lithium selective membranes. a) Illustration of ZIF‐8, HKUST‐1, and UiO‐66 structures, and their pore sizes. Reproduced with permission.^[^ ^113^ ^]^ Copyright 2020, Elsevier. b) Schematic of ion separation process through a ZIF/GO/AAO membrane and the crystal structure of ZIF‐8. Reproduced with permission.^[^ ^114^ ^]^ Copyright 2018, American Association for the Advancement of Science. c) Schematic illustration of ZIF‐8/MPPM preparation. Reproduced with permission.^[^ ^115^ ^]^ Copyright 2023, MDPI. d) Schematic of TA─Fe III/ZIF‐8 fabrication on polypropylene (PP) membrane and test setup. Reproduced with permission.^[^ ^116^ ^]^ Copyright 2020, Elsevier. e) Illustration of UiO‐66 membrane by cathodic deposition. Reproduced with permission.^[^ ^117^ ^]^ Copyright 2023, Elsevier.

Fortunately, MOF materials represented by ZIF‐8,^[^ ^110^ ^]^ UiO‐66,^[^ ^111^ ^]^ and HKUST‐1,^[^ ^112^ ^]^ due to their angstrom‐sized windows and nanometer‐sized cavities, have been proven to effectively separate Li^+^ ions by utilizing the differences in dehydration energy (**Figure** **10**a). These materials, which exhibit excellent stability in aqueous environments, are considered to have significant potential.

A major task is to efficiently combine MOF materials with membranes. To date, polycrystalline MOF membranes (PMOFs) and MMMs are the two most commonly used methods.^[^ ^118^ ^]^ PMOF membranes are continuous MOF selective layers prepared on porous carriers by solvothermal, secondary growth, and other methods. The substrate material not only provides sites for the growth of the MOF layer, but also effectively improves the mechanical stability of the composite membrane. Zhang et al.^[^ ^114^ ^]^ prepared ultrathin ZIF‐8 membrane with the assisted growth of nanoporous graphene oxide (GO) layer on an aluminum oxide (AAO) support (Figure 10b). *I*–*V* measurements of single‐ion solutions showed that lithium ions exhibited the highest transport rate (Li^+^ (4.6) > Na^+^ (3.4) > K^+^ (2.1) > Rb^+^ (1.0)), which could be attributed to the partial dehydration effect of ZIF‐8 structure. Instead of an inorganic substrate, Zhao et al.^[^ ^115^ ^]^ introduced a large number of hydroxyl and amine groups on the surface of microporous polypropylene membranes (MPPMs) and constructed a homogeneous and defect‐free ZIF‐8 layer through solvothermal method (Figure 10c). The ion‐permeable flux of ZIF‐8/MPPM was 0.1171 mol m^−2^ h^−1^ in a 0.1 m single‐salt solution test, with selectivity of 1.83 and 8.99 for Li^+^/Na^+^ and Li^+^/Mg^2+^, respectively. Although there is a slight decrease in a binary salt solution, the selectivity still remained at 1.34 and 5.63. Mohammad et al.^[^ ^116^ ^]^ adopted a simpler approach of using a multifunctional tannic acid and iron complex (TA─Fe III) as an intermediate layer to assist the growth of ZIF‐8 membranes instead of using an expensive PDA. *I*–*V* tests of 1 m single‐salt solutions showed good separation of univalent and multivalent ions (Figure 10d). Xie et al.^[^ ^117^ ^]^ recently reported an innovative method for the preparation of continuous UiO‐66 membrane by cathodic deposition (Figure 10e). The UiO‐66 membrane deposited on AAO exhibited selective ion transport performance with a Li^+^/Mg^2+^ selectivity of 286 and a lithium permeance of 11.2 mol m^−2^ h^−1^ in the *I*–*V* test of 1 m single‐salt solution. PMOF membranes exhibit notable selective separation capabilities by directly utilizing the angstrom‐sized pores inherent to MOF layers. In addition, this biochannel‐like multilayer structure also ensures efficient ion transport. However, the complex preparation processes and structures not only directly pose challenges such as membrane–substrate adhesion concerns, grain boundary defects, and mechanical stability of crystals, but also affect economic viability and scalability considerations for industrial production.

**Figure 11:**
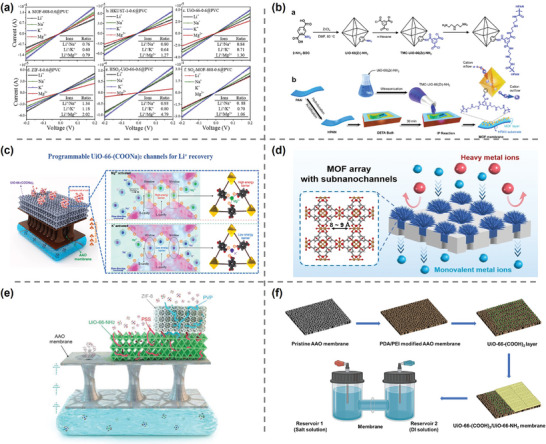
Illustration of ZIF‐8, HKUST‐1, UiO‐66, and their pore sizes. a) *I*–*V* curves of MOFs@PVC membrane. Reproduced with permission.^[^ ^113^ ^]^ Copyright 2020, Elsevier. b) Reaction for the synthesis of TMC‐functionalized UiO‐66(Zr)─NH_2_ nanoparticles and the fabrication of membrane. Reproduced with permission.^[^ ^119^ ^]^ Copyright 2020, Elsevier. c) Illustration of UiO‐66─(COONa)_2_ membranes with programmable ion channels and its working mechanism. Reproduced with permission.^[^ ^120^ ^]^ Copyright 2023, Elsevier. d) Illustration of MOF array membranes with sub‐nanochannels. Reproduced with permission.^[^ ^121^ ^]^ Copyright 2023, John Wily and Sons. e) Illustration of MOF‐on‐MOF asymmetrical architecture. Reproduced with permission.^[^ ^122^ ^]^ Copyright 2022, John Wiley and Sons. f) Schematic of membrane synthesis and test in a H‐cell. Reproduced with permission.^[^ ^123^ ^]^ Copyright 2022, Elsevier.

Another approach is to introducing MOF as a filler into the polymer matrix is to prepare MMM.^[^ ^118^ ^]^ This method offers a more stable structure and controllable processability. Zhang et al.^[^ ^113^ ^]^ directly mixed four MOF materials and two MOF derivatives with PVC. The hybrid membranes were prepared through solution casting method. ZIF‐8, which has the smallest pore size, showed the best selectivity among original MOF‐based membranes at a content of 60%. The sulfonated MOF showed an improved separation ability, which could be attributed to the difference in the ion transport rates caused by the sulfonic acid groups and the enhanced electrostatic repulsion produced by the stacked ions (**Figure** **11**a). Xu et al.^[^ ^119^ ^]^ grafted trimethylacetyl chloride (TMC) on UiO‐66(Zr/Ti)─NH_2_. The modified MOF was successfully immobilized in the polyamide layer synthesized on the surface of hydrolyzed polyacrylonictile (HPAN) through interface polymerization with diethylenetriamine (DETA) (Figure 11b). The composite membrane exhibited excellent separation efficiency for monovalent and divalent ions (Na^+^/Mg^2+^ = 13.44, Li^+^/Mg^2+^ = 11.38).

MOFs can be further functionalized or prepared with special structures to increase their selectivity and permeability. Xiao et al.^[^ ^120^ ^]^ innovatively synthesized UiO‐66─(COONa)_2_ membranes with programmable ion channels (Figure 11c). The affinity difference between the carboxyl groups on the modified MOF and different ions leads to the change of ion channels dimension. This controllable pore size exhibits intelligent ion permeability and selectivity. Wu et al.^[^ ^121^ ^]^ deposited ZIF‐8 into the nanoporous defects of a hierarchical defect layer material membrane, resulting in a synergistic 2D membrane with a Li^+^ flux of 1.73 mol m^−2^ h^−1^ and a selectivity of Li^+^/Mg^2+^ up to 31.9 (Figure 11d). Similarly, Wu et al.^[^ ^121^ ^]^ filled HKUST‐1 into the micropores of polyethylene terephthalate (PET) to enhance the ionic selectivity, and obtained up to 3930 ± 373 Li^+^/Zr^4+^ selectivity and Li^+^ flux of 1.97 mol h^−1^ m^−2^. Inspired by the asymmetry of biological ion channels, Abdollahzadeh et al.^[^ ^122^ ^]^ designed a asymmetric membrane with a MOF‐on‐MOF double‐layer structure (Figure 11e). The membrane showed ionic current rectification effects similar to diodes. Energy barriers formed by sudden changes in cavity size disrupt the hydration structure of ions, making them more prone to be captured by nucleophilic substances within the MOF. Among the three MOF‐on‐MOF structures constructed, UiO‐66─NH_2_(PSS)/ZIF‐8(PVP) showed the highest separation capacity. A similar structure was reported by Xiao et al.,^[^ ^123^ ^]^ where a UiO‐66─(COOH)_2_/UiO‐66─NH_2_ double‐layer MOF structure demonstrated excellent Li^+^/Mg^2+^ separation performance in experiments with natural brine environments (Figure 11f).

MOF‐based membranes demonstrate notable separation ability for both monovalent and multivalent ions. However, in MMMs, filler agglomeration and compatibility issues between MOFs and the polymer matrix can limit filler loading, resulting in suboptimal separation performance. The stability and processability of the polymer matrix need to be considered. Additionally, the innovative biomembrane‐like asymmetric structure has shown significant performance improvements, providing valuable inspiration for future related research.

### 4.4 Membranes Based on Other Materials

**Figure 12:**
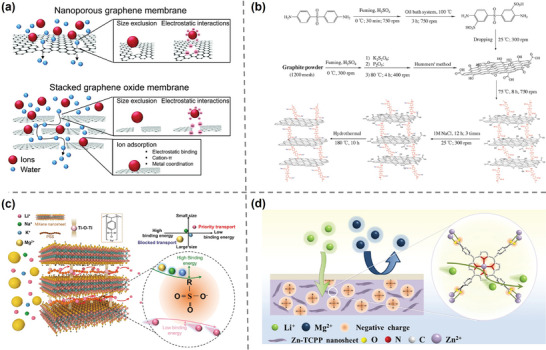
Other materials for lithium recovery. a) Schematic of two types of GO membranes with ion permeability and selectivity as well as their separation mechanism. Reproduced with permission.^[^ ^124^ ^]^ Copyright 2015, Royal Society of Chemistry. b) Schematic of the synthesis and structure of rGO─SDDS─rGO ion‐selective membranes. Reproduced with permission.^[^ ^126^ ^]^ Copyright 2018, John Wiley and Sons. c) Illustration diagram of fast transport sub‐nanochannels in MXene/PSS membrane. Reproduced with permission.^[^ ^129^ ^]^ Copyright 2021, John Wiley and Sons. d) Diagrammatic sketch of PVA‐modified SPES‐based lithium‐ion selective membrane. Reproduced with permission.^[^ ^130^ ^]^ Copyright 2022, Elsevier.

In addition to MOF, there are some other 2D materials have been designed for Li^+^ separation. For example, graphene and its derivatives are considered highly promising materials due to their unique single‐atom thickness, 2D structure, excellent chemical stability, and flexibility. While pristine graphene is impermeable to water and ions, researchers have explored modified graphene‐based materials, including nanoporous graphene sheets and stacked GO (**Figure** **12**a).^[^ ^124^, ^125^ ^]^ Zhao et al.^[^ ^126^ ^]^ grafted sulfonated 4,4′‐diaminodiphenyl sulfone (SDDS) onto GO sheets and reduced them in a hydrothermal reactor to form rGO─SDDS─rGO membranes (Figure 12b). The stacked GO membranes have abundant sulfonic acid groups and tunable layer spacing, which can be precisely controlled through the chain length of SDDS. The multilayer structure with an average layer spacing of about 0.48 nm is effective in selectively transporting lithium ions through dehydration differences and exhibits permeation selectivity of 5.27 and 4.72 for Li^+^/Mg^2+^ and Li^+^/Ca^2+^, respectively, in ED tests. The same research group^[^ ^127^ ^]^ constructed multilayer framework membranes by chemical self‐assembly using graphene oxide and sulfonated amino–polystyrene nanospheres with ample amino and sulfonate groups (rGO@SAPS). The selective separation efficiency parameters of this membrane for Mg^2+^/Li^+^ and K^+^/Li^+^ were 46.13% and 9.90%, respectively, within 20 min in an electrodialysis system. Ahmadi et al. developed a membrane with an asymmetric structure by modulating the morphology and chemistry of GO nanochannels. This membrane has an “energy surge baffle” effect and shows a significantly increased Li^+^ selectivity and flux.^[^ ^128^ ^]^

MXene is a novel family of 2D materials that has attracted widespread attention from researchers in fields such as energy, catalysis, and environmental conservation due to its high conductivity and antibacterial capabilities.^[^ ^131^ ^]^ Despite the nanoscale spacing of stacked MXene membranes, swelling in aqueous environments or under external voltages is a significant impediment to their ion separation capabilities. Lu et al.^[^ ^129^ ^]^ introduced poly(sodium *p*‐styrene sulfonate) with sulfonate sites into stacked MXene nanosheets to make a lamellar membrane with Li^+^ selectivity (Figure 12c). The self‐cross‐linked MXene micro‐interlayer channels and sulfonate group transport sites with different ionic affinities worked together to obtain ultrahigh Li^+^ selectivity. This multilayer structured stacking approach, despite its good Li^+^ separation capability, still faces the challenges of precise control of layer spacing and instability caused by external driving forces or water environment.

In addition, the cross‐linked polymer may also provide spatial hindrance to inhibit transport with larger ions. Tao et al.^[^ ^130^ ^]^ performed surface cross‐linking of hydroxyl‐rich polyvinyl alcohol (PVA) onto sulfonated polysulfone (SPES) membranes (Figure 12d). The dense PVA/glutaraldehyde (GA) cross‐linked layer provided spatial hindrance, resulting in a 2.65‐fold increase for the selectivity of Li^+^/Mg^2+^. To address issue of the sharp decrease in ion flux, negatively charged Zn─tetrakis(4‐carboxyphenyl)porphyrin (TCPP was incorporated. At a content of 0.6%, the PVA/SPES/Zn─TCPP membrane exhibited optimal performance (*J*_Li+_ = 9.12 × 10^−9^ mol cm^−2^ s^−1^, Mg^2+^/Li^+^ selectivity = 8.99).

Constructing single‐ion channel apertures to exclude ion based on size or hydration is considered to be the most direct and efficient strategy for improving selectivity. In addition to separation performance, stability and recovery efficiency are also crucial factors. While numerous studies have demonstrated the effectiveness of lithium‐specific materials, most still lack the economic feasibility for large‐scale industrial production and long‐term stability in complex aqueous environments, which are crucial issues that need to be addressed in future research.

## 5 Electrostatic Repulsion

In addition to adjusting pore size to accommodate the size of ions or hydrated shell, the exploitation of disparities in ionic charge has also been considered as a viable strategy to enhance ion selectivity.^[^ ^132^, ^133^ ^]^ For Li^+^/Mg^2+^ separation, size exclusion proves may not be appropriate, while electrostatic repulsion provides a feasible solution. Positively charged membranes are anticipated to facilitate the separation of monovalent/multivalent ions due to the Donnan exclusion effect.^[^ ^134^ ^]^ Introducing the charge within membranes required the rational design of material structures. In this section, we focus on the membrane modifications with positively charged groups, and their results in ion separations and Li^+^ recovery.

### 5.1 Amine‐Based Modification

The lone electron pair of the nitrogen atom of simple amines intends to combine with H^+^ in aqueous condition to form the ammonium ion at a weakly acidic or neutral pHs, thereby enhancing water solubility and acquiring a positive charge. The electrically charged properties of amines have been demonstrated and applied in biology, water treatment, and other fields.^[^ ^135^ ^]^ In terms of membrane recovery, polymeric amines such as polyethyleneimine (PEI) have been extensively studied, especially with respect to the positively charging modification of NF membranes. Moreover, some other amine‐based polymers or small molecules have also been reported to have useful functionality.

#### 5.1.1 Modification of Nanofiltration Membranes

NF is a membrane‐based separation technique with molecular weight cutoff range of 200–1000 Da and pore size of 0.5–2 nm. Although NF have achieved notable success in both ion separation research and industrial production due to their unique separation mechanisms, most commercial NF membranes, prepared via interfacial polymerization (IP) between piperazine (PIP) and trimesoyl chloride, still exhibit limited Li^+^ recovery capacity.^[^ ^57^ ^]^ The residual acyl chloride tends to hydrolyze to generating negatively charged carboxyl groups, while membranes with positive charge are of greater interest for monovalent/multivalent ion separation.^[^ ^136^, ^137^ ^]^ Consequently, modification of NF membrane to introduce positive charges has received much interest.

**Figure 13:**
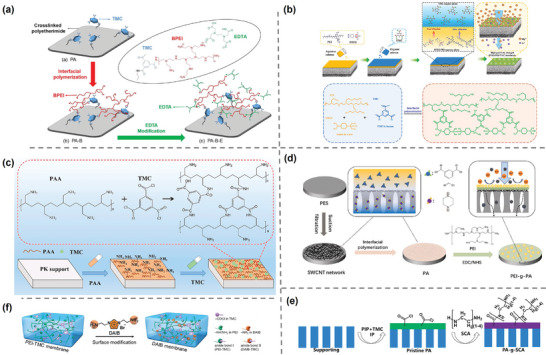
Amino‐group‐contained modification of NF membrane. a) Schematic of the preparation of EDTA‐modified NF membrane. Reproduced with permission.^[^ ^138^ ^]^ Copyright 2017, Elsevier. b) Schematic of fabrication of the PAA/TMC NF membrane. Reproduced with permission.^[^ ^140^ ^]^ Copyright 2023, Elsevier. c) Schematic representation of the preparation, separation process, and chemical reaction of DTES/PEI/TMC hybrid membrane. Reproduced with permission.^[^ ^141^ ^]^ Copyright 2023, Elsevier. d) Schematic diagram of the preparation process of positively charged NF membranes with dual‐skin layer. Reproduced with permission.^[^ ^143^ ^]^ Copyright 2021, Elsevier. e) Illustration of DAIB‐modified PEI─TMC membrane. Reproduced with permission.^[^ ^144^ ^]^ Copyright 2021, John Wiley and Sons. f) Schematic of the preparation of PA‐*g*‐SCA membrane. Reproduced with permission.^[^ ^145^ ^]^ Copyright 2023, Elsevier.

One approach is to modify the membrane with amine‐rich precursors. Li et al.^[^ ^138^ ^]^ prepared NF membranes by replacing conventional PIP with branched polyethyleneimine (BPEI) (**Figure** **13**a).The abundant amine groups in BPEI were protonated in water to increase the positive charge on the membrane surface. The NF membranes were further modified with ethylenediaminetetraacetic acid (EDTA), which has the ability to trap divalent ions, and showed enhanced separation performance and excellent stability. Similarly, Xu et al.^[^ ^139^ ^]^ used PEI with long branched polymeric chains to react with TMC on polyethersulfone substrates. The obtained membrane with positively charged surface was tested in a solution with an ion concentration of 2000 ppm and achieved a separation factor for Li^+^/Mg^2+^ of 20. Xu et al.^[^ ^140^ ^]^ used polyallylamine (PAA) with a higher amine content to prepare NF membrane with a highly positive charged surface though an IP reaction with TMC (Figure 13b). The NF membrane with enhanced electrostatic repulsion effect showed a retention rate of 99.1% for Mg^2+^ and separation factor for Li^+^/Mg^2+^ up to 82.3_._ This premodified method is not only effective in enhancing the positive charge, but also easy to synthesize and has good stability. However, due to the higher molecular weight of PEI, the diffusion rate is slower during the polymerization process, resulting in the formation of thick layers with nonuniform pore size and reduced permeability.

Efforts have been made to improve permeability and water flow. Wu et al.^[^ ^141^ ^]^ introduced a monomeric amine silane coupling agent (3‐diamino‐methyl‐cyclohexyl triethoxysilane (DTES)) into PEI/TMC membrane. DTES not only modifies SiO_2_ with high hydrophilicity in situ, but its diamino group can also react with the acyl chloride group of TMC to ensure high compatibility of SiO_2_ nanoparticles (Figure 13c). The prepared membranes exhibit higher porosity and hydrophilicity. Guo et al.^[^ ^142^ ^]^ prepared “double Janus” PES/carboxylated cellulose nanocrystal (CNC─COOH)/polyamide (PA) NF membranes with hydrophobic/hydrophilic units and both positive and negative charges. The large amount of negative charged carboxyl groups in the interlayer appeared to improve not only water flux, but also the positive potential of PA layer due to the increase of unreacted ─NH_2_, leading to enhanced ion separation.

The depletion of amine groups during the IP process in the reaction with TMC leads to a decreased final surface positive charge density of the membrane. Therefore, researchers have explored the introduction of positive charges through surface modification. Reactants containing amine or hydroxyl groups can be used to modify the surface of the NF membrane, which proved to be an economical and effective strategy. Yang et al.^[^ ^143^ ^]^ developed a dual‐layer NF membrane by grafting branched PEI onto the surface of the traditional PIP/TMC NF membrane with single‐walled carbon‐nanotube‐coated PES as the support (Figure 13d). The synergistic effect of the size exclusion of the polyamide layer and the electrostatic repulsion of the PEI layer resulted in 98.5% exclusion of MgCl_2_ and a Li^+^/Mg^2+^ separation factor of up to 33.5 in simulated brines. Lu et al.^[^ ^147^ ^]^ used PEI with much lower molecular weight for secondary nonaqueous interfacial reaction to construct positively charged nanolayers on nascent PA NF membranes. The membrane achieved a Li^+^/Mg^2+^ separation factor of 12.37 in simulated brine at a concentration of 2000 ppm and a Mg^2+^/Li^+^ mass ratio of 150, which is 2.6 times higher than that of unmodified PA membranes. These experimental results not only demonstrated the feasibility of surface grafting modification to increase the positive membrane charge, but also showed that modifiers with smaller molecular weights could form a denser grafting network, resulting in a higher charge density.

Small molecule ammonium salts with more flexible sizes have been designed and synthesized for surface modification of NF membranes. Peng and Zhao^[^ ^144^ ^]^ developed a novel electrolyte monomer containing a bidentate amine group (diaminoethimidazole bromide salt, DAIB) for the surface modification of PA membranes (Figure 13e). The positively charged DAIB not only effectively excludes Mg^2+^ ions, but also results in a 5 times increase of the permeability of the membrane due to its high hydrophilicity. Gu et al.^[^ ^148^ ^]^ used a polymeric quaternary ammonium salt (3,5‐dimethylhydrazide‐benzyl trimethyl ammonium bromide) to surface modify the PEI/TMC polyamide membrane. The modified membrane's surface charge density increased to +5.16 mC m^−2^, nearly 3 times that of the initial PA membrane, and achieved a separation factor of 60.1 in a 2000 ppm Mg^2+^/Li^+^ mixed solution (mass ratios of MgCl_2_/LiCl = 20). Li et al.^[^ ^145^ ^]^ used short‐chain amino‐rich monomers (SCA, H(NHCH_2_CH_2_)_1–4_─NH_2_)) for membrane surface modification and fabricated more uniformly positively charged NF membranes (Figure 13f). Promisingly, surface modification using ammonia‐rich precursors achieves higher positive charge graft density as well as controllable membrane thickness without affecting the size exclusion effect of the original NF membrane.

Based on a similar principle, Si et al.^[^ ^149^ ^]^ embedded aminated covalent organic framework (COF) nanosheets into polyamides to decrease the internal density and enhance positive charge to the membranes. The resulting membranes exhibited high water permeability and high Li^+^/Mg^2+^ selectivity of 19.6 L m^−2^ h^−1^ bar^−1^. Notably, Wang et al.^[^ ^146^ ^]^ synthesized a nonpolyamide nanofiltration membrane by Cu^2+^‐assisted Cu─*m*‐phenylenediamine (MPD) self‐polymerization on a PES substrate. Copper cations and protonated amino groups together provided a positive surface charge. This pH‐responsive NF membrane exhibited high water permeability of 16.2 ± 2.7 Liter/m^2^/h (LMH) bar^−1^ and Li^+^/Mg^2+^ selectivity of 8.0 ± 1.0 under acidic conditions, with excellent permeability selectivity balance and biofouling resistance.

#### 5.1.2 Modification of Other Membranes

**Figure 14:**
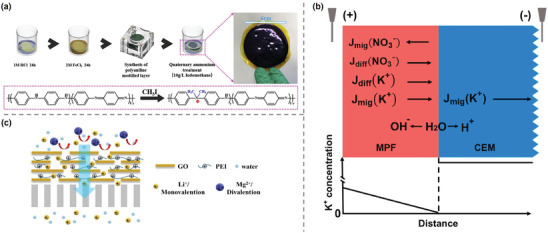
Amino‐group‐contained modification of other membrane. a) Schematic of synthesis of quaternized polyaniline membrane. Reproduced with permission.^[^ ^150^ ^]^ Copyright 2020, Elsevier. b) Interface between multilayer polyelectrolyte film (MPF)/cation‐exchange membrane (CEM). Reproduced with permission.^[^ ^153^ ^]^ Copyright 2022, Elsevier. c) Mechanism of the separation for PEI membranes. Reproduced with permission.^[^ ^152^ ^]^ Copyright 2023, American Chemical Society.

In addition to NF membranes, a similar approach can be used to modify other types of membranes. Pang et al.^[^ ^150^ ^]^ carried out in situ polymerization and deposition of polydopamine on the surface of sulfonated polysulfone, followed by a quaternization reaction with iodomethane to prepare composite membranes with tunable positive charge surfaces (**Figure** **14**a). The prepared membranes exhibited enhanced Na^+^/Mg^2+^ permeation selectivity of 4.1 and Li^+^/Mg^2+^ permeation selectivity of 1.75 in a simulated mixed‐brine system. Ding et al.^[^ ^151^ ^]^ used Nafion membrane as the substrate material and coated its surface with (PAH/PSS)_5_PAH, resulting in K^+^/Mg^2+^ and Li^+^/Mg^2+^ selectivity >100 (Figure 14b). Wang et al.^[^ ^152^ ^]^ constructed GO─PEI membranes with positively charged channels by link‐branching GO nanosheets with polyelectrolyte polyethyleneimine (PEI) molecules (Figure 14c). The synergistic effect of size repulsion and electrostatic repulsion enabled the GO─PEI membranes to display a high selectivity of 22.2 for Li^+^/Mg^2+^ and a competitive Li^+^ permeation rate of 0.09 mol m^−2^ h^−1^ in a binary permeation test. Similarly, Huang et al.^[^ ^153^ ^]^ used positively charged PEI molecules to adjust the interlayer charges and structure of GO membranes, creating charged interlayer channels in the GO membrane. The optimized GO─PEI membrane with a thickness of about 100 nm exhibited a Li^+^ permeation rate of 0.306 mol m^−2^ h^−1^ and a Li^+^/Mg^2+^ selectivity of 21.9.

As noted above, the separation of Li^+^ from multivalent ions can be improved by introducing a positive charge on the membrane. Although this strategy does not enhance the separation between monovalent ions, it can still be used as an effective auxiliary method for the lithium recovery. The premodification with amine‐rich precursors, although simple and structurally stable, does not allow control of the grafting charge and can affect the structure and porosity of the membranes. In addition, surface modification is subject to problems of graft stability and large‐scale production. Therefore, improving modification methods to combine enhanced stability and controllability should be the main focus for the future research.

### 5.2 Other Membrane Modifications to Introduce Positive Charge

**Figure 15:**
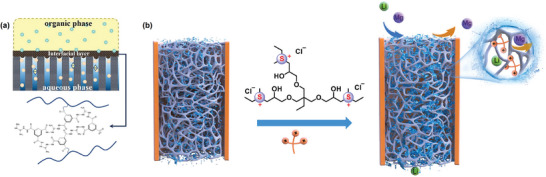
Modifications to achieved enhanced positively charged membranes. a) Illustration of the formation of the interfacial layer. Reproduced with permission.^[^ ^154^ ^]^ Copyright 2023, Elsevier. b) Modification of PEI─TMC NF membrane with a hydroxyl‐containing sulfonium. Reproduced with permission.^[^ ^155^ ^]^ Copyright 2023, Elsevier.

In addition to the commonly used amine‐based modification, several other types of functional groups have also been employed for modifications to introduce positive charge. Zhang et al.^[^ ^154^ ^]^ utilized 1,3‐diaminoguanidine hydrochloride as a monomer to synthesize a polyamide composite membrane through interface polymerization with TMC (**Figure** **15**a). Guanidine carries a strong positive charge under neutral or strong alkaline conditions, which can be imparted to a composite membrane. This modification resulted in excellent multivalent ion suppression capability and Li^+^ selectivity. Peng et al.^[^ ^155^ ^]^ were the first to employ thiol‐based modifications for nanofiltration membranes. Hydroxyl‐containing thiols were used to modify PEI─TMC membranes through “hydroxyl–acetyl” condensation, imparting a positively charged layer. It was suggested that the effective introduction of positive charges improved the membrane's loose structure, enhanced lithium selectivity, and positively affected its performance (Figure 15b).

While limited research exists regarding the positively charge modification through nonamine‐based groups, increasingly innovative methods have facilitated the study of Li^+^ recovery based on electrostatic repulsion. Enhanced positive charge of membrane has proved its efficiency in separating ions with similar hydrodynamic volumes but differing charges. Therefore, the development of simple, stable, and controllable method of preparing membranes enriched with positive charges is an important direction for future research.

## 6 Conclusion and Future Work

**Figure 16:**
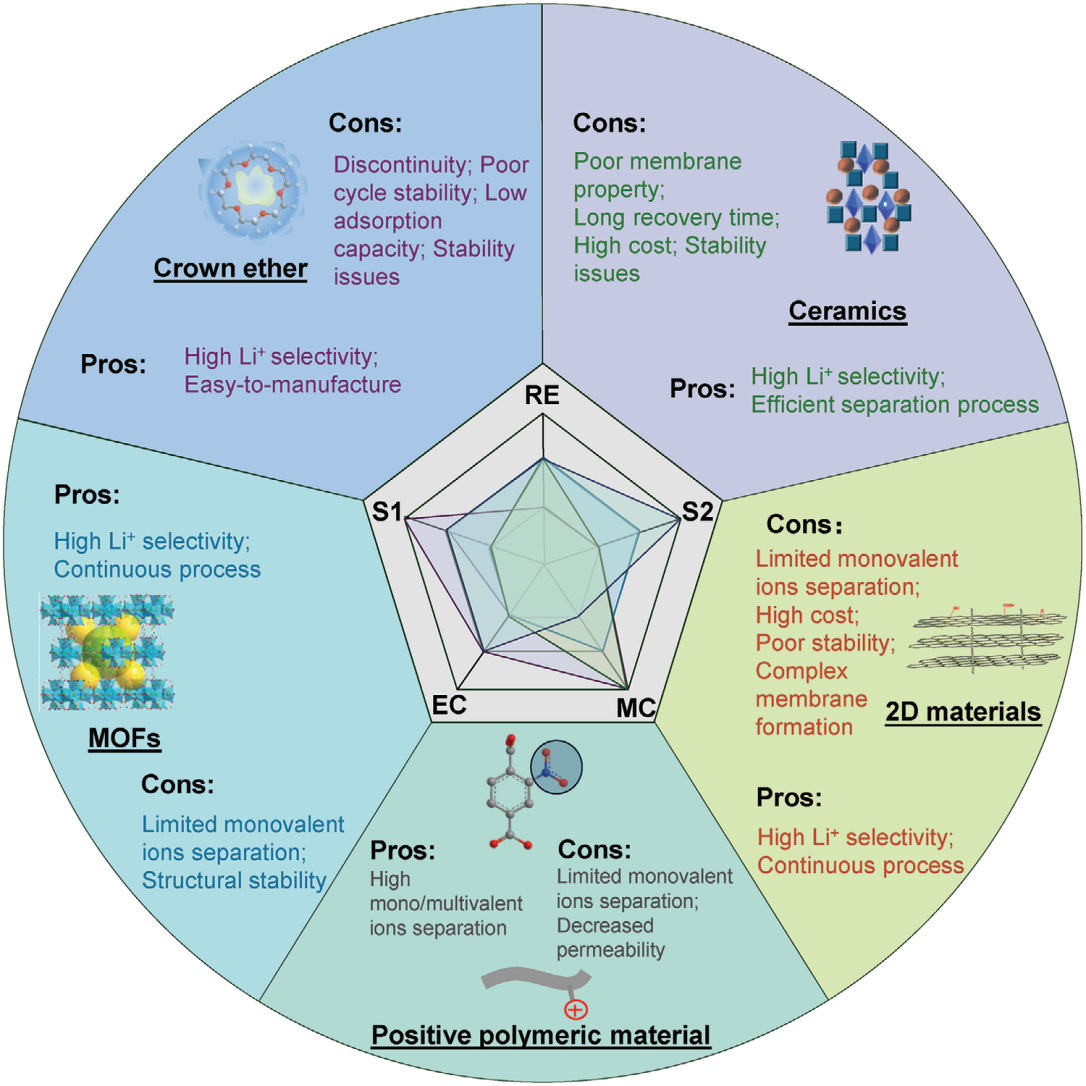
Brief summary of the key cutting‐edge materials for Li recovery. MOFs: metal–organic frameworks. S1: selectivity. S2: stability. RE: recovery efficiency. EC: energy consumption. MC: material cost.

This article reviews and discusses the separation mechanism, evaluation metrics, and latest research of Li^+^ selective membranes from both theoretical and practical aspects. Size exclusion and electrostatic effects are considered two key principles in lithium recovery membrane design. By utilizing the differences of ionic size and charge of ions/hydration ions, they have demonstrated excellent separation capabilities for monovalent/multivalent ions and even between different monovalent ions. This review summarizes recent representative materials and studies based on these principles, several of which are highly innovative and inspiring (**Figure** **16**). In general, CE‐based membranes have demonstrated excellent selective lithium recovery effects, particularly when the CE membranes are used for permeation separations, which effectively address the discontinuity of the adsorption process while maintaining selectivity. However, the challenge of effectively integrating CEs with membranes remains a significant issue. Ceramic‐based membranes have shown remarkable selectivity, but their poor mechanical properties pose a major obstacle to industrial application. While MOF‐based membranes exhibit reasonable ion separation capabilities, they are still insufficient for practical use. Although some 2D‐material‐based membranes are highly innovative, their high costs and complex manufacturing processes will significantly limit their development. Positively charged modified nanofiltration membranes have shown promising Li^+^/Mg^2+^ separation capabilities but have limited efficacy in separating monovalent ions. Additionally, this review article provides a critical summary of the methods and standards used for various evaluation tests.

It is clear that membrane process development for lithium recovery is still in its early stage. Although this review describes significant progress and has achieved some success at the laboratory scale (TRLs 3–5), several key issues still need to be addressed before their future industrial application. First, although some of the currently reported membranes exhibit greatly improved recovery efficiency and selectivity, they are still far from meeting the requirement for industrial applications, especially in the separation of low‐concentration and nonequimolar ions from real lithium sources, which are still to be investigated. Second, membrane stability is a critical concern. Many current tests suffer from short duration or limited cycles and fail to simulate real‐world conditions. The complex preparation processes and expensive materials used for many membranes pose challenges for large‐scale commercial production. Furthermore, considerations of energy consumption and cost remain important factors that need to be addressed.

Constructing stable membrane pores at the sub‐nanometer scale remains a difficult challenge. Most of the current studies mainly focuses on existing microporous structures such as CEs, MOFs, and other materials. Therefore, despite the inherent difficulties, exploring new structures with the capability for monovalent ion separation at the angstrom scale is both useful and necessary. On the other hand, developing membranes with uniform pore size distribution, good stability, and ease of preparation poses another significant challenge. In addition to the traditional polymer and inorganic membranes, the development of composite membranes has provided new inspirations for the research. Encouragingly, some comprehensive studies on the transport mechanisms and separation processes of solutes, especially ions, in sub‐nanochannels have been reported in recent years and research in this area has growing momentum. The authors suggest that future work could focus on the following areas.

1. Although constructing membranes with angstrom‐degree control of porosity is still extremely challenging, materials with specific lithium‐ion selective structures (e.g., crown ethers, ceramics, and MOFs) have demonstrated ability for lithium‐ion recovery and separation. Constructing stable membrane pores at a sub‐nanometer scale has also been proven by introducing rigid units into flow‐battery membranes,^[^ ^27^, ^157^, ^158^, ^159^, ^160^ ^]^ these knowledge can be adopted to design highly permselective Li‐recovery membranes. Therefore, we recommend effective utilization and further exploration of the materials with well‐defined sub‐nanometer porosity.
2. Bioionic semipermeable membranes remain by far the best performing membranes with extremely high ion selectivity and transport rates. The asymmetric structure of bioionic semipermeable membranes has inspired researchers and promises an optimal balance of selectivity, permeability, and stability, thus suggesting a promising model for materials and process development.
3. The electrical charge of ions provides a natural advantage over gases and liquids in the separation process. Electrodialysis promises to be a very simple and effective method for the recovery of lithium with excellent recoveries at lower energy consumption and shorter time if suitably selective ED membranes can be developed.

## Conflict of Interest

The authors declare no conflict of interest.
